# Stable Isotopes Reveal Long-Term Fidelity to Foraging Grounds in the Galapagos Sea Lion (*Zalophus wollebaeki*)

**DOI:** 10.1371/journal.pone.0147857

**Published:** 2016-01-25

**Authors:** Massimiliano Drago, Valentina Franco-Trecu, Luis Cardona, Pablo Inchausti, Washington Tapia, Diego Páez-Rosas

**Affiliations:** 1 Programa PROMETEO-SENESCYT, Secretaría de Educación Superior, Ciencia, Tecnología e Innovación, Quito, Ecuador; 2 Department of Ecology & Evolution, Centro Universitario Regional Este (CURE), University of the Republic (UdeLaR), Maldonado, Uruguay; 3 Department of Ecology & Evolution, Faculty of Sciences, University of the Republic (UdeLaR), Montevideo, Uruguay; 4 Department of Animal Biology, Faculty of Biology, University of Barcelona, Barcelona, Spain; 5 Department of Applied Research, Galapagos National Park Service, Puerto Ayora, Galápagos, Ecuador; 6 Galapagos Conservancy, Santa Cruz, Galápagos, Ecuador; 7 Universidad San Francisco de Quito (USFQ) and Galapagos Science Center, San Cristóbal, Galápagos, Ecuador; 8 Dirección Parque Nacional Galápagos, Unidad Técnica Operativa San Cristóbal, Galápagos, Ecuador; Norwegian Polar Institute, NORWAY

## Abstract

Most otariids have colony-specific foraging areas during the breeding season, when they behave as central place foragers. However, they may disperse over broad areas after the breeding season and individuals from different colonies may share foraging grounds at that time. Here, stable isotope ratios in the skull bone of adult Galapagos sea lions (*Zalophus wollebaeki*) were used to assess the long-term fidelity of both sexes to foraging grounds across the different regions of the Galapagos archipelago. Results indicated that the stable isotope ratios (δ^13^C and δ^15^N) of sea lion bone significantly differed among regions of the archipelago, without any significant difference between sexes and with a non significant interaction between sex and region. Moreover, standard ellipses, estimated by Bayesian inference and used as a measure of the isotopic resource use area at the population level, overlapped widely for the sea lions from the southern and central regions, whereas the overlap of the ellipses for sea lions from the central and western regions was small and non-existing for those from the western and southern regions. These results suggest that males and females from the same region within the archipelago use similar foraging grounds and have similar diets. Furthermore, they indicate that the exchange of adults between regions is limited, thus revealing a certain degree of foraging philopatry at a regional scale within the archipelago. The constraints imposed on males by an expanded reproductive season (~ 6 months), resulting from the weak reproductive synchrony among females, and those imposed on females by a very long lactation period (at least one year but up to three years), may explain the limited mobility of adult Galapagos sea lions of both sexes across the archipelago.

## Introduction

All otariid species are colonial breeders that may reach locally high population densities at breeding sites [1]. Females of colonially breeding species with dependent offspring often forage in the vicinity of rookeries and behave as central place-foragers [2, 3]. According to the optimal foraging theory, central-place foragers are more likely to accept patches close to the central place, even when they provide a relatively low net rate of energy gain [4, 5]. This is because of the increasing traveling costs to foraging grounds with distance, which must be compensated by increased energy gain to be rewarding [6, 7]. In this scenario, fidelity to foraging areas at the colony level may be the proximate cause of separation of foraging areas by nearby colonies, provided that individual consumers consistently detect features of local foraging areas such as changes in sea temperature or local productivity across successive trips [8, 9]. Telemetry studies revealed that many central-place forager seabird species have strong fidelity to foraging areas among individuals from the same breeding site [10, 11] and colony-specific foraging areas have also been documented for different central-place forager otariids such as Antarctic fur seals (*Arctocephalus gazella*) [12, 13], South American sea lions (*Otaria flavescens*) [14, 15] and northern fur seals (*Callorhinus ursinus*) [16, 17].

After the breeding season, otariids may disperse over broad areas and individuals from different rookeries may potentially share the same feeding grounds at that time. Unfortunately, information about the patterns of habitat use of most otariids after the breeding season is scarce because satellite tags usually last for only a few months [18]. Stable isotope analysis constitutes an alternative approach to study patterns of habitat use and diet at several spatial-time scales [18], and offers valuable insights into the behavior of otariids after the breeding season (e.g. [19, 20]).

The tissue isotopic composition of consumers in marine systems is ultimately set by the isotopic composition of the assimilated food. Since these inputs can show spatial isotopic gradients, consumer isotopic data can be used to study, amongst other things, their spatial variation in their trophic habits [18]. While nitrogen isotope ratios increase in a predictable way along trophic chains, allowing comparisons of consumer trophic position [21, 22], carbon isotope ratios mirror baseline ecosystem signatures and provide information on foraging strategies and feeding locations [18, 23]. The time period over which stable isotopes reveal useful information on feeding habits depends on the biochemical turnover rate of the tissue, because stable isotope turnover rates in tissues vary as a function of the tissue metabolic rate [24]. As a consequence, tissues with high turnover rates provide information on the diet assimilated from recent feeding bouts, while tissues with lower turnover rates provide information on diet assimilated from more remote feeding bouts [24].

Bone tissue, due to its relatively slow turnover, constitutes a long-term integrator (~5 years) of isotope ratios and a moderator of sporadic isotopic fluctuations, which makes it useful for comparing the foraging habits and feeding regimes among individuals over a long period of time [25–27]. However, the use of bone tissue samples from individuals belonging to scientific collections can have the disadvantage of working with a limited sample size, impossible to increase especially in the case of threatened and protected species. Furthermore, stable isotope analysis from bone tissue is affected by some drawbacks common to other stable isotope analyses. For instance, they provides less detailed information on dietary composition than stomach content or scat analysis and cannot be used to determine the depth of the foraging habitat, such as time-depth recorders do, or replace data (i.e. migratory routes and geographical position) obtained by satellite telemetry. Accordingly, caution is needed when interpreting stable isotope data and all the results derived from isotopic analyses should be considered as an approximation due to the multiple assumptions and limitations involved.

The Galapagos sea lion (*Zalophus wollebaeki*) is an endemic otariid species that breeds on almost all islands of the archipelago, with the highest density of individuals at the central and southern islands. This species, maybe in adaptation to or because of the relatively low productivity of the marine environment, is the smallest of all sexually dimorphic sea lions, with adult males having a maximum weight of about 158 kg and a standard length of 205 cm and with adult females of a maximum weight of about 95 kg and a length of 176 cm [28]. Its overall population, currently estimated around 16,000 individuals [29], has suffered a drastic reduction over the last 30 years [30, 31] that lead the IUCN to classify it as endangered [32]. This generalist species feeds on a wide variety of both benthic and pelagic prey, whose abundance varies both temporally and geographically [33, 34].

Using pup fur isotopic values as proxies of the habitat use of their mothers, female Galapagos sea lion were shown to exploit foraging grounds closer to their rookeries during the first months of the pupping season [35]. The studied breeding sites of the Galapagos sea lion are separated by distances ranging from 52 to 238 km [35]. The maximum recorded distance travelled by a Galapagos sea lion in a foraging trip is 42 km [36–38], which suggest that female Galapagos sea lions from distant rookeries are unlikely to use the same foraging grounds. However, the constraints on the overall duration and distance travelled by females while foraging due of pup rearing do not apply to males which might scatter and forage all over the archipelago once the breeding season is over [39]. Furthermore, females may disperse and move to the most productive foraging grounds in the western part of the archipelago as pups grow and their swimming skills improve [40]. Thus, the use of distinct foraging grounds by females during the pupping season may not apply to males or even to females in other periods of the year.

Here, we use stable carbon and nitrogen isotopes of bone tissue from adult Galapagos sea lions to assess the long-term fidelity of both sexes to foraging grounds across the Galapagos archipelago. We hypothesize that differences in isotopic contents could reflect either large-scale oceanographic contrasts in productivity in Galapagos, or the high and persistent specialization of Galapagos sea lion population to trophic resources that are locally abundant in certain parts of the archipelago. This information is useful to identify management units within the Galapagos archipelago.

## Materials and Methods

### Ethics Statement

Ethical approval was not required for this study because it was conducted on Galapagos sea lion skulls coming from scientific collection. All necessary permits were obtained for the described study, which complied with all relevant regulations. The sampling and exporting of all skull bone samples of Galapagos sea lions were authorized by the Galapagos National Park Service under the permit No 184/2013 PNG.

### Study Area and Sample Collection

**Fig 1:**
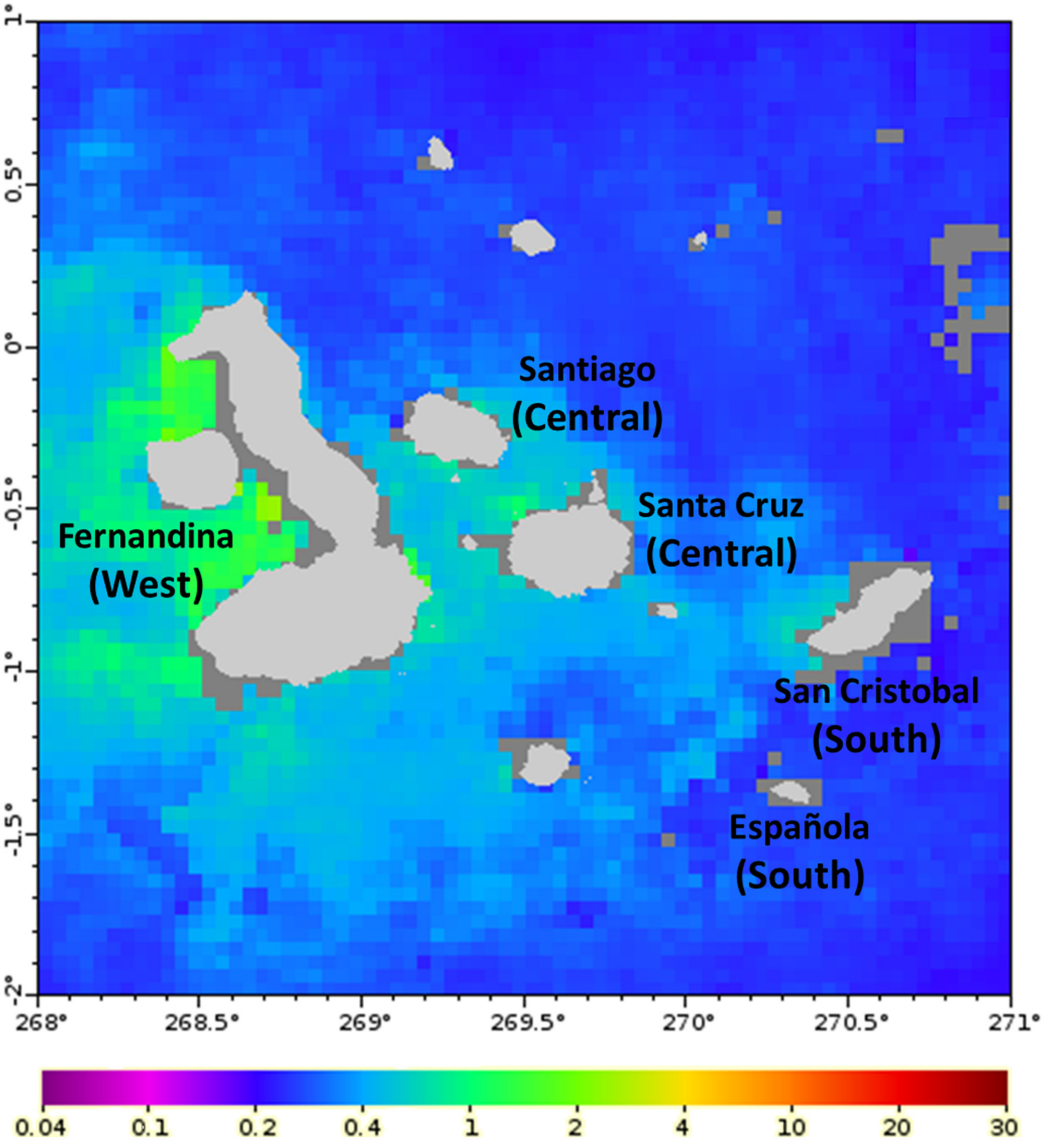
Map of the Galapagos archipelago showing the islands where sea lion skulls were collected and the chlorophyll levels. Chlorophyll values are the cumulative average values of chlorophyll-a concentration (mg/m^3^) from 1 September 1997 to 31 August 2001 derived from SeaWiFS Project (http://oceancolor.gsfc.nasa.gov). The hydrogeographic regions, in agreement with Ruttenberg et al.[42], are denote by the names in brackets.

The regional biogeography of the Galapagos archipelago proposed by Harris [41] and Ruttenberg et al. [42] was considered to identify potential spatial foraging segregation for the Galapagos sea lion. Accordingly, bone samples were collected from adult Galapagos sea lions of both sexes found dead in breeding sites of five islands (Fernandina, San Cristóbal, Española, Santa Cruz and Santiago; Fig 1), corresponding to three contrasting hydrogeographic regions of the Archipelago in terms of both sea temperature and productivity [43]: West, dominated by the Cromwell Current with the coldest sea surface temperatures and higher productivity; South, dominated by the Humboldt Current where sea surface temperatures are cool but warmer than the West; and Central, a mixed region where sea surface temperatures are similar or slightly warmer than the South (Fig 1). Both primary productivity and marine species diversity decrease from the western to the southern regions of the Galapagos archipelago [44].

All bone samples (West region: 7 males and 9 females; Central region: 11 males and 16 females; South region: 15 males and 16 females; Table 1) were obtained from the skulls of the scientific collection of the Charles Darwin Research Station at Santa Cruz Island (Galapagos). Skulls selected for sampling were restricted to Galapagos sea lions that were died from 2000 to 2001 to minimize any confounding effects due to long-term changes in the stable isotope baseline (Table 1). Furthermore, although the exact age of a sampled individual was unknown to us, only skulls of physically mature adult specimens were considered to avoid any possible age-related bias [45]. Sex was determined based on external morphology (e.g. presence of baculum bone in males) during sample collection and eventually assessed using other secondary sexual characteristics of the skull [46]. The bone sample from the skull of each individual that was used for the isotopic analysis consisted of a small fragment of turbinate bone taken throughout its entire thickness from the nasal cavity. This bone type was selected because it is relatively easy to crush and its sampling does not damage the skull for any subsequent studies. All skull bone samples were cleaned with distiller water to remove impurities and stored dry until analysis.

**Table 1:** Skulls of Galapagos sea lions investigated in the present study.

Sample location		Female					Males				
Region	Island	Sample ID	Dead year	δ^15^N (‰)	δ^13^C (‰)	C/N (%)	Sample ID	Dead year	δ^15^N (‰)	δ^13^C (‰)	C/N (%)
West	Fernandina	233	2001	13.2	-12.9	3.2	2409	2001	12.6	-12.5	3.3
West	Fernandina	2387	2001	12.0	-12.5	3.1	2416	2001	14.4	-12.7	3.1
West	Fernandina	2397	2001	15.2	-14.2	3.3	2278	2001	13.3	-12.7	3.2
West	Fernandina	2407	2001	12.2	-13.3	3.1	2311	2001	12.6	-12.4	3.2
West	Fernandina	2411	2001	12.9	-12.6	3.2	2313	2001	12.8	-12.6	3.2
West	Fernandina	2414	2001	13.8	-13.3	3.2	2428	2001	11.8	-13.5	3.2
West	Fernandina	2417	2001	12.2	-12.9	3.1	2484	2001	12.7	-12.8	3.2
West	Fernandina	2427	2001	12.1	-12.6	3.2					
West	Fernandina	2393	2001	13.5	-13.2	3.0					
**Mean ± SD**				**13.0 ± 1.0**	**-13.0 ± 0.5**				**12.8 ± 0.7**	**-12.7± 0.4**	
Central	Santiago	164	2001	13.4	-13.1	3.1	54	2001	13.5	-13.1	3.0
Central	Santiago	165	2001	12.1	-12.9	3.2	2497	2001	12.7	-13.6	3.2
Central	Santiago	168	2001	14.9	-12.2	3.1					
Central	Santa Cruz	2336	2000	11.8	-13.6	3.1	2445	2000	12.2	-13.7	3.2
Central	Santa Cruz	2338	2000	11.2	-13.4	3.2	2448	2000	13.0	-13.9	3.3
Central	Santa Cruz	2288	2000	12.2	-13.9	3.1	2460	2000	11.7	-13.5	3.2
Central	Santa Cruz	2304	2000	11.4	-13.3	3.3	2486	2000	14.6	-13.7	3.5
Central	Santa Cruz	2316	2000	13.0	-13.4	3.4	2297	2000	11.8	-13.4	3.1
Central	Santa Cruz	2464	2000	12.4	-13.4	3.3	2449	2000	11.6	-13.5	3.2
Central	Santa Cruz	2469	2000	12.2	-13.3	3.0	2462	2000	11.8	-13.6	3.4
Central	Santa Cruz	2470	2000	12.8	-13.7	3.1	2385	2001	11.4	-13.7	3.1
Central	Santa Cruz	2337	2001	12.6	-13.2	3.2	2455	2001	14.9	-13.5	3.4
Central	Santa cruz	2343	2001	13.1	-14.2	3.1					
Central	Santa Cruz	2395	2001	11.4	-13.6	3.1					
Central	Santa Cruz	2434	2001	12.8	-13.5	3.2					
Central	Santa Cruz	2352	2001	11.6	-13.5	3.2					
**Mean ± SD**				**12.4± 0.9**	**-13.4± 0.4**				**12.7± 1.2**	**-13.6± 0.2**	
South	San Cristóbal	2451	2000	12.3	-13-5	3.1	2465	2000	12.5	-13.3	3.1
South	San Cristóbal	2289	2001	13.0	-14.1	3.2	2404	2001	12.3	-13.3	3.1
South	San Cristóbal	2324	2001	11.4	-13.5	3.1	2420	2001	11.3	-13.3	3.2
South	San Cristóbal	2390	2001	11.8	-13.7	3.2	2421	2001	11.7	-13.8	3.0
South	San Cristóbal	2396	2001	11.9	-13.7	3.2	2480	2001	11.7	-13.5	3.1
South	San Cristóbal	2413	2001	12.3	-13.6	3.3	2485	2001	12.3	-13.6	3.2
South	San Cristóbal	2468	2001	11.6	-13.6	3.4	2481	2001	12.2	-13.9	3.1
South	Española	2281	2000	11.6	-13.7	3.2	1684	2000	11.5	-13.7	3.2
South	Española	2447	2000	11.9	-13.6	3.2	1686	2000	11.8	-13.6	3.2
South	Española	2471	2000	11.7	-13.8	3.1	2326	2000	11.7	-13.9	3.3
South	Española	2488	2000	12.9	-14.1	3.6	2452	2000	11.4	-13.5	3.2
South	Española	2410	2001	11.3	-13.8	3.2	2454	2000	11.9	-13.7	3.2
South	Española	2389	2001	13.6	-13.1	3.1	2399	2001	12.0	-13.4	3.0
South	Española	2415	2001	13.2	-12.8	3.3	2406	2001	13.8	-13.7	3.1
South	Española	2426	2001	12.4	-14.1	3.1	2394	2001	11.6	-13.0	3.1
South	Española	2443	2001	11.7	-13.3	3.1					
**Mean ± SD**				**12.2± 0.7**	**-13.6± 0.4**				**12.0± 0.6**	**-13.5± 0.3**	

δ^13^C: stable carbon isotope values;δ^15^N: stable nitrogen isotope values; C/N: carbon to nitrogen mass ratios;Bold isotopic values: mean ± SD for each sex within each region;Dead year: year of collection; Sample size: only one sample from each skull was analyzed; Age: all specimens are adult physically mature sea lions of unknown age.

### Stable Isotope Analyses

Following initial sampling, cleaning and preparation, the skull bone fragments from each individual were dried at 6°C and grounded into a fine powder using a mortar and pestle. Lipids were removed from each sample using a chloroform-methanol (2:1) solution [47] because lipids are depleted in ^13^C compared with other molecules and variability in lipid content of samples may result in undesirable variability in δ^13^C values [23]. Nevertheless, given that the chemical lipid extraction may lead to unpredictable changes in δ^15^N values due inter alia to the inadvertent removal of amino acids [48, 49], we extracted lipids for carbon isotope analysis and used a non-extracted subsample for nitrogen determination. Furthermore, as bone samples contain a high concentration of inorganic carbon that may add undesirable variability to δ^13^C [50], the subsamples used for carbon isotope analysis were also previously treated by soaking in 0.5 N hydrochloric acid (HCl) for 24 h to decarbonise them [51].

Approximately 1 mg of each powdered and processed sample of bone was weighed into tin capsules (3.3 × 5 mm) and analyzed by elemental analysis isotope ratio mass spectrometry(EA-IRMS), using a model FlashEA 1112 elemental analyzer (ThermoFisher Scientific, Milan, Italy) coupled with a Delta C isotope ratio mass spectrometer (ThermoFinnigan, Bremen, Germany). Analyses were performed at the Scientific-Technical Services of the University of Barcelona, Spain.

Stable isotope abundances, expressed in delta (δ) notation, where the relative variations in stable isotope ratios are expressed in per mil (‰) deviations from predefined international standards, were calculated as:

δjX = [(Xj/Xi)sample(Xj/Xi)standard]–1

where ^j^X is the heavier isotope (^13^C or ^15^N), and ^i^X is the lighter isotope (^12^C or ^14^N) in the analytical sample and in the international measurement standard [52]; reference standards were the Vienna Pee Dee Belemnite (VPDB) calcium carbonate for δ^13^C and atmospheric nitrogen (air) for δ^15^N. Secondary isotopic reference materials of known ^13^C/^12^C ratios, as given by the International Atomic Energy Agency (IAEA, Vienna, Austria), were used for calibration at a precision of 0.05‰. These include polyethylene (IAEA CH_7_, δ^13^C = –32.1 ‰), L-glutamic acid (IAEA USGS_40_, δ^13^C = -26.4‰) and sucrose (IAEA CH_6_, δ^13^C = –10.4‰). For nitrogen, secondary isotopic reference materials of known ^15^N/^14^N ratios, namely (NH_4_)_2_SO_4_ (IAEA N_1_, δ^15^N = +0.4‰ and IAEA N_2_, δ^15^N = +20.3‰), L-glutamic acid (IAEA USGS_40_, *δ*^15^N = -4.5‰) and KNO_3_ (IAEA NO_3_, δ^15^N = +4.7‰), were used to a precision of 0.2‰. All these secondary isotopic reference materials were employed to recalibrate the system and compensate for any measurement drift over time once every 12 samples analyzed.

### Data Analyses

We quantified the carbon to nitrogen (C/N) mass ratio of each analyzed sample as a control for the data quality (e.g. adequate lipid extraction) of the bone collagen [18]. Furthermore, before all other statistical analyses, we verified the normality of the data by means of the Lilliefors test, and its homoscedasticity by means of the Levene test. Separate two-way ANOVAs (since δ^13^C and δ^15^N were uncorrelated) were used to assess the differences in the average δ^13^C and δ^15^N values depending on gender and region and their interactions, and the Scheffe test was used for the post-hoc comparisons.

SIBER (Stable Isotope Bayesian Ellipses in R) [53] was used to define the isotopic niche space among Galapagos sea lions from the three different regions as a measure of their isotopic resource use area at the population level. This method is a Bayesian version of Layman metrics [54] that unlike of the Euclidean methods (e.g. convex hulls), can incorporate uncertainties such as sampling biases and small sample sizes into niche metrics [53]. Based on Markov-Chain Monte Carlo (MCMC) simulation, this approach assigns measures of uncertainty to construct parameters of ellipses in a way similar to a bootstrap. We used standard ellipse areas corrected for small sample size (SEA_C_) to represent the Galapagos sea lions from the three different regions in the isotopic space and estimated the width of their isotopic niche using the Bayesian standard ellipse areas (SEA_B_). The latter captures all the same properties as SEAc, but it is unbiased with respect to sample size and exhibits more uncertainty with smaller sample size [53]. Furthermore, we calculated the magnitude of the isotopic overlap among sea lions from the three different regions based on 100,000 posterior draws of the SEA_C_ parameters [53].

Data are always shown as mean ± standard deviation (SD), unless otherwise stated, and the significance level considered for all tests was 0.05 [55]. All statistical analyses were carried out in free software R 3.0.2 [56] and all codes for SIBER analyses were contained in the library SIAR (Stable Isotope Analysis in R) [57].

## Results

**Fig 2:**
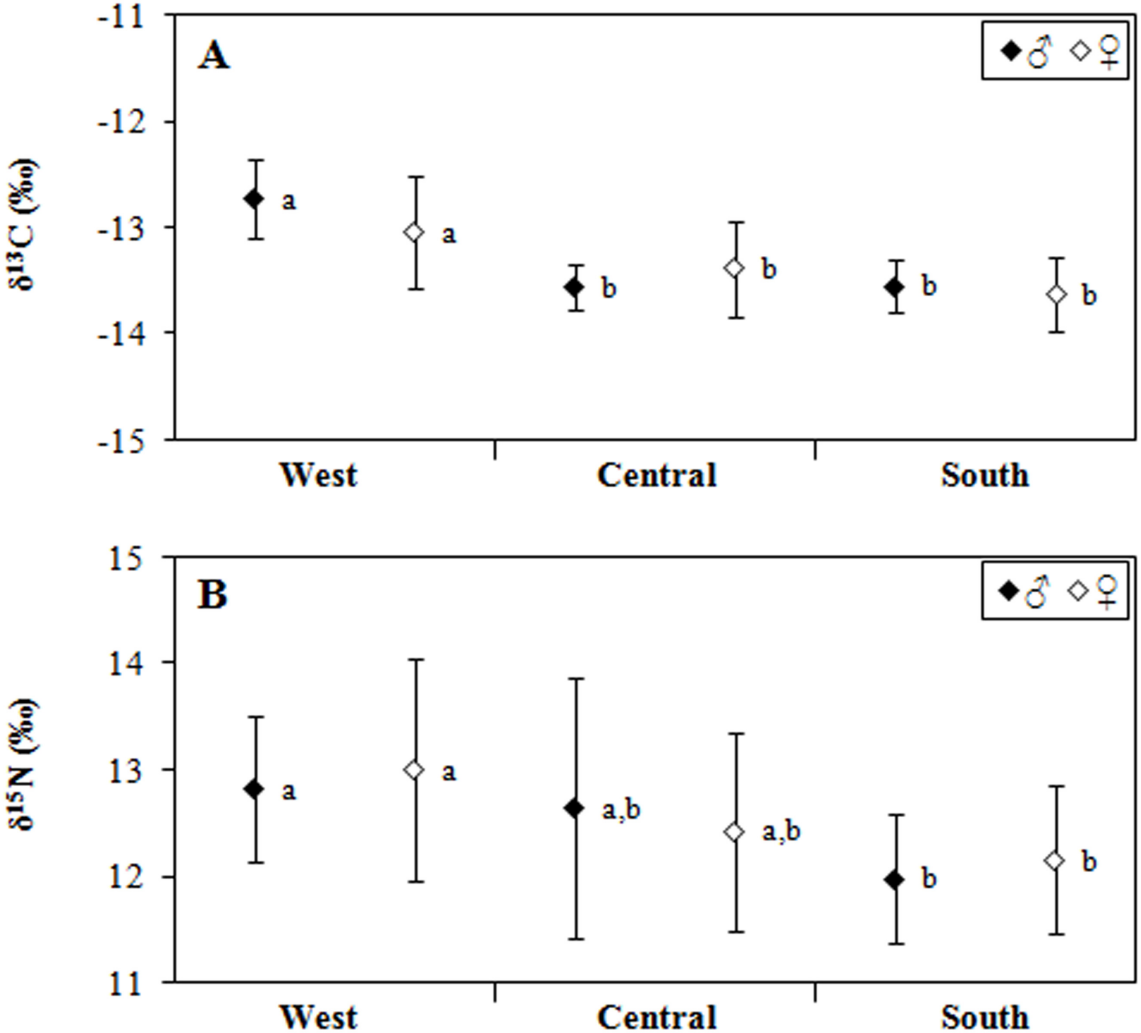
Mean (±SD)values of δ^13^C (A) and δ^15^N (B) for bone from the skulls of male and female sea lions collected in three different regions of the Galapagos archipelago. Regions with different superscript (lower-case letters) are statistically different in their mean values according to the Scheffe's post hoc test. Vertical bars show standard deviation.

Table 1 shows the stable isotope (δ^13^C and δ^15^N) values and the carbon to nitrogen (C/N) mass ratios of the Galapagos sea lions from the different islands and regions of the Galapagos archipelago. The C/N mass ratio of all samples ranged from 3.0 to 3.6 (Table 1), well within the theoretical range that characterizes unaltered bone collagen protein [58]. The stable isotope values of sea lion bone significantly differed among regions (Two-way ANOVA; δ^13^C: F_2,68_ = 19.603, p < 0.001; δ^15^N: F_2,68_ = 4.832, p = 0.011), and there was no significant differences between sexes (Two-way ANOVA; δ^13^C: F_1,68_ = 0.635, p = 0.428; δ^15^N: F_1,68_ = 0.085, p = 0.771). Further, the interaction between sex and region was not statistically different for either δ^13^C (F_2,68_ = 2.610, p = 0.081) or δ^15^N (F_2,68_ = 0.262, p = 0.771). Post-hoc tests revealed statically significant differences between sea lions from the western and southern regions for both δ^13^C and δ^15^N (Fig 2). The average δ^13^C of the sea lions from the southern region was not different from that of the sea lions from the central region and the average δ^15^N of the sea lions from the central region was not different from that of the sea lions from the other two regions (Table 1 and Fig 2).

**Fig 3:**
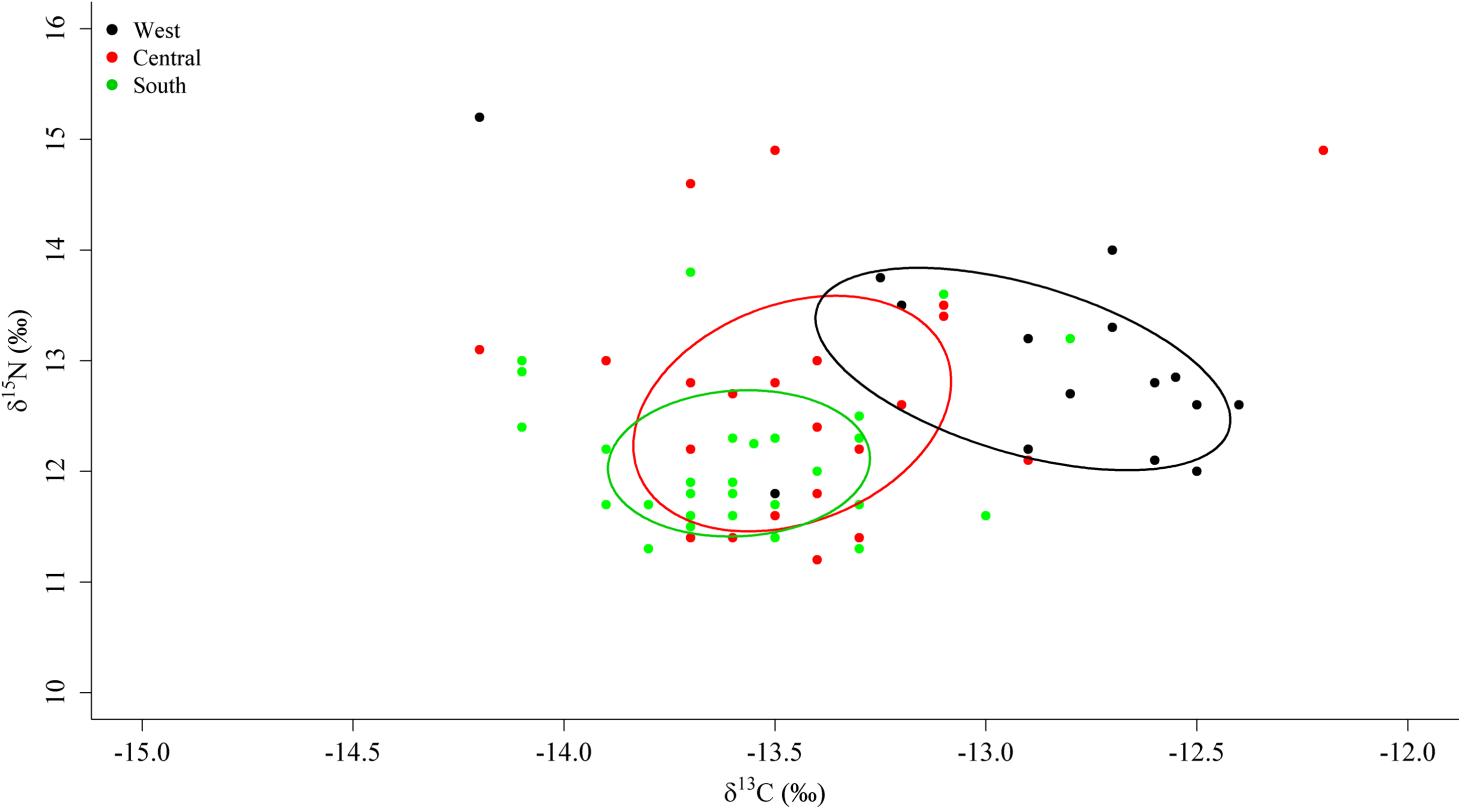
Area of the isotopic niche of Galapagos sea lions in the three different regions of the archipelago. Ellipse areas (solid lines)calculated with SEA_C_ using the bivariated isotopic values (solid circles) of males and females from each region(West, Central and South).

The Bayesian ellipse of sea lions from the south (SEA_B_ mean = 0.87 ‰^2^, 95% credibility interval of 0.58 to 1.19 ‰^2^) and west (SEA_B_ mean = 1.59 ‰^2^, 95% credibility interval of 0.88 to 2.43 ‰^2^) regions did not overlap (Fig 3), hence confirming different resource use patterns for these two groups of sea lions. On the other hand, the Bayesian ellipse of sea lions from the central region (SEA_B_ mean = 1.51 ‰^2^, 95% credibility interval of 0.99 to 2.11 ‰^2^) overlapped with those of sea lions from the other two regions (Fig 3). The overlap area (0.57 ‰^2^) of the Bayesian ellipses from the central and south regions represented the 47.0% of the ellipse surface of the former and the 88.5% of the ellipse surface of the latter. Conversely, the overlap area (0.24 ‰^2^) of the Bayesian ellipses of sea lions from the central and west regions represented only the 19.7% of the ellipse surface of the former and the 19.5% of the ellipse surface of the latter (Fig 3).

## Discussion

The results reported here suggest a certain degree of foraging philopatry at a regional scale within the archipelago for both sexes of the Galapagos sea lions, as far as a west-south gradient in the stable isotope baseline exists in the archipelago. Changes in the stable isotope ratios of sea lion prey within the archipelago have not been specifically investigated to our knowledge, but available information (Table 2) shows a weak west-south decrease in both the δ^13^C and δ^15^N values of jacks (Carangidae) and sardines (Clupeidae). Furthermore, stable isotope ratios in suckling pups of the Galapagos sea lions revealed a similar geographic pattern [35], which agrees with the pattern reported here for adults.

**Table 2:** Stable isotope values (mean ± SD) of the potential Galapagos sea lion prey from the three different regions of the archipelago.

Scientific name	Family	Habitat	Region	Sampling date	n	δ^13^C (‰)	δ^15^N (‰)	Source
*Selar crumenophthalmus*	Carangidae	Pelagic	West	2008	5	-15.9 ± 0.3	11.9 ± 0.9	[59]
*Selar crumenophthalmus*	Carangidae	Pelagic	South	2008	3	-16.5 ± 0.5	11.2 ± 0.6	[60]
*Sardinops sagax*	Clupeidae	Pelagic	West	2008	8	-16.8 ± 0.7	9.7 ± 1.7	[59]
*Opisthonema berlangai*	Clupeidae	Pelagic	Central	2008	2	-17.1 ± 0.1	9.6 ± 0.7	[60]
*Opisthonema berlangai*	Clupeidae	Pelagic	South	2008	4	-17.4 ± 0.2	9.5 ± 0.5	[60]

Regional differences in the isotope baseline within the Galapagos Islands are certainly small but the consistent pattern across fishes and sea lion pups and adults reinforces their biological significance and the gradient observed in the δ^15^N and δ^13^C values is consistent with the primary productivity pattern in the archipelago (Fig 1). The oceanic areas adjacent to the Galapagos Islands experience open ocean conditions and are characterized by relatively low δ^15^N variability and values at the base of the food web [61]. Conversely, upwelling results into higher δ^15^N values [62], because ^15^N is recycled more quickly, and higher δ^13^C values, because of the high consumption of ^12^C by phytoplankton due to a high primary productivity [63, 64]. Hence, differences in the stable isotope ratios of sea lions along the productivity gradient just reveal foraging phylopatry.

A potentially confounding factor is the capacity of Galapagos sea lions to forage either pelagically or benthically [36, 37] which might obscure the regional differences in the δ^13^C values if a strong pelagic-benthic gradient existed and the proportion of pelagic and benthic foragers varied along the productivity gradient. Although other studies have shown that the Galapagos sea lion have generalist diets whose species composition did not significantly differ between the southern and western regions [35, 65], the possibility that foraging strategies vary along the productivity gradient deserves further research.

Our results then would render support to the hypothesis that there is a long-term fidelity of the Galapagos sea lions to foraging habitats close to their breeding grounds within large areas of the archipelago, beyond what reported by satellite tracking at short time-scale [36, 37]. Although not always close to breeding sites, long-term foraging site fidelity has also been observed in other otariid species, such as the Australian sea lion (*Neophoca cinerea*) [66], Australian fur seal (*Arctocephalus pusillus doriferus*) [20] and Antarctic fur seal [67, 68]. Our conclusion is also supported both by the typically short distances that the Galapagos sea lion travel (41.76 ± 20.27 km, range: 14.3 to 76 km) [36] during their foraging trips and by detected differences in isotopic contents in lactating Galapagos sea lion females from different regions during gestation [35]. However, all conclusions stemming from scat analyses, diving behavior, satellite telemetry or the isotopic analyses of tissues with high turnover rates could only convey information on a short-term (days to a few months) basis. By being a long-term integrator (~5 years) that smoothes out the annual isotopic variability in highly dynamic oceanographic conditions, the bone tissue used in the study can provide a substantial characterization of the contrasts in the trophic ecology of the Galapagos sea lion in different parts of the archipelago, including for the first time information about adult males.

It should be noted, however, that stable isotope analysis from bone has also some limitations. First, and as a direct consequence of a low isotopic turnover rate, the stable isotope ratios reported here may be influenced in some individuals not only by the adult stage diet but also by the diet at the juvenile/immature period. Since (i) ontogenetic foraging changes are common in otariids [45, 69] and (ii) they mainly disperse at young ages [70–72], it is likely that the bone value in young adults is a mixture of various isotopic habitats and diets. A second drawback is that bone sampling is highly intrusive and hence samples are usually collected only from dead stranded specimens or from scientific collections. That certainly limits sample size and may result into insufficient power to capture the actual individual levels of variability within the population. Here we have analyzed all the samples available at the Charles Darwin Research Station, statistical models were validated by residual analyses and the final models had a very good fit to the available data. Perhaps, a larger sample size would reveal differences between the central and southern populations, but we are confident that the available data set confirms, at least, limited exchange between the western part of the Galapagos Islands and the remaining of the archipelago.

Otariids are income breeders and females alternate feeding trips to the sea with suckling bouts on land during the long lactation period (approximately one year) when pups almost exclusively feed on maternal milk [73]. Pups that remain unattended while their mothers are at sea may die of starvation [74, 75]. Under the constraints of lactation, otariid females would be expected to modify their foraging behavior (prey choice and time spent at sea) to shorten the foraging trip durations while they are feeding pups more or less continuously and to meet their energy requirements [76]. As a result of these trade-offs, otariid females are expected to forage closer to the rookery, particularly during the early lactation [13, 77, 78], dispersing to more productive and distant foraging grounds as pups improve their swimming skills and certainly after weaning. However, the lactation period of Galapagos sea lions is much longer than that of most otariids since mothers generally wean their offspring after a long period lasting at least one and usually two or even three years [79]. This behavior is according to the seasonal-productivity hypothesis, which predicts an inverse correlation between latitude (a proxy for oceanic productivity) and lactation length in pinnipeds [80], and implies that new pups are born while the older siblings of different cohorts are still being nursed [79]. In the case of the Galapagos sea lion females, the need to minimize traveling costs and to feed regularly their several pups can be considered a continuous pressure throughout each female’s life and may lead to philopatric tendencies found in this study. The constraints related to the long duration of pup dependency do not apply for Galapagos sea lion males that could scatter and forage anywhere once the breeding season is over.

Contrary to what happens for most otariids, which aggregate into large colonies only during a few weeks in late spring or early summer for mating immediately after that females give birth [73, 81], for Galapagos sea lions the rutting period lasts for approximately six months [28, 38], with certain variation depending on the island [39]. This is because of the weak reproductive synchrony of females, which may give birth and come into estrus at any time from August to January [28, 38]. Only the males of the Galapagos sea lion and those of the Australian sea lion face similar challenging long breeding seasons [73] which make impossible to any male continuously to defend a territory and monopolize and mate receptive females by remaining fasting at the rookery during such a long time span [82]. Australian sea lion males respond to this challenging behavior as central place foragers and using the same foraging grounds year round [66]. A similar situation is likely to be true for the Galapagos sea lion, at least during the six months of their extended rutting period.

Thus, the constraints imposed by the long dependency of several pup cohorts on females and by the long duration of the reproductive season on males may explain both the spatial foraging segregation and the philopatric tendencies of both sexes of the Galapagos sea lion [79, 83]. In this context, when resource availability is unpredictable, as in tropical marine environments with low seasonality, individuals foraging area fidelity can provide ecological benefits, such as resources and predation risk knowledge [84].

The two constraints (long pup dependency and reproductive season) may also explain the absence of long-term sexual segregation of the Galapagos sea lion foraging habits that could be expected for sexually dimorphic species [45, 85, 86]. This is contrary to what is expected for a sexually dimorphic air-breathing marine predator, where large-bodied males should be able to travel longer distances from the rookeries and to exploit larger three-dimensional habitats than females [87] and where such differences are expected to be consistent through time [20, 88]. It should be noted, however, that sexual size dimorphism is much smaller in the Galapagos sea lion than in other otariids and hence it is not so surprising that males and females exploit similar foraging habitats (depths and distances from the rookery) and consume prey at similar trophic levels [39]. Furthermore, the relationships among body size, diving performance and isotopic niche are not necessarily constant and individual traits other than size, such as energy requirements or breeding constraints, as well as variations in population density and prey availability play a fundamental role on determining resource partitioning between the two sexes. For instance, the males of the highly sexually dimorphic South American sea lion can certainly dive deeper and longer than females [14] and both sexes differ in their isotopic niche during the early years of their adult live but share the same isotopic niche at an older age [45]. Furthermore, isotopic niches have historically converged as the population from northern Patagonia recovered from commercial sealing and the per capita food availability changed [26]. On the other hand, males and females share the same isotopic niche for most of the year in Uruguay and Brazil, but differences emerge immediately before the breeding season [89]. In any case, it should be kept in mind that the isotopic niche is just a proxy of the actual trophic niche and the absence of differences in stable isotope ratios does not necessarily mean identical trophic niches. Further research based on stomach content analysis is required to be sure that male and female Galapagos sea lions do not differ in trophic niche.

In conclusion our results show that: i) spatial trophic segregation occurs in Galapagos sea lions, ii) the males can also display foraging philopatry, iii) local trophic resources are essential for the breeding success and positive growth of colonies in different islands of the archipelago, iv) this apex predator could be used as an “ecosystem sentinel” since changes in the quality or integrity of the foraging zones could be used as an indicator of environmental degradation and of declines in the abundance or diversity of its trophic resources in Galapagos [90, 91], all of which are key monitoring features of this Biosphere Reserve (UNESCO 1985) and World Natural Heritage Site (since 1978).
